# Thoracolumbar/Lumbar Degenerative Kyphosis—The Importance of Thoracolumbar Junction in Sagittal Alignment and Balance

**DOI:** 10.3390/jpm14010036

**Published:** 2023-12-27

**Authors:** Chenjun Liu, Rile Ge, Haoyuan Li, Zhenqi Zhu, Weiwei Xia, Haiying Liu

**Affiliations:** 1 Department of Spinal Surgery, PeKing University People’s Hospital, 11th. Xizhimen South Ave., Beijing 100044, China; liuchenjun@bjmu.edu.cn (C.L.); 2211210325@stu.pku.edu.cn (H.L.); zhuzhenqi@pkuph.edu.cn (Z.Z.); 2 Chinese Preventive Medicine Association, Committee on Prevention and Control of Spinal Disease, Gulou West Street No. 154, Beijing 100009, China; 3 Trauma Medicine Center, PeKing University People’s Hospital, 11th. Xizhimen South Ave., Beijing 100044, China; gerile@pkuph.edu.cn

**Keywords:** thoracolumbar/lumbar degenerative kyphosis, thoracolumbar junctional kyphosis, sagittal balance, proximal junctional kyphosis

## Abstract

Purpose: To conduct a more comprehensive study of sagittal alignment in patients with thoracolumbar/lumbar (TL/L) degenerative kyphosis. Methods: A total of 133 consecutive patients from September 2016 to March 2019 with degenerative spinal kyphosis were enrolled. These patients were divided into different types according to sagittal alignment, including thoracolumbar junctional kyphosis (TLJK). Then, we divided the patients with TLJK into two groups: the Sagittal Balance group (C7-SVA < 50 mm) and the Sagittal Imbalance group (C7-SVA ≥ 50 mm). The sagittal parameters of each type or group were compared and correlation analysis was conducted. Results: Thoracolumbar/lumbar degenerative kyphosis consists of four types: Type I, lumbar kyphosis; Type II, degenerative flat back; Type III, thoracolumbar junctional kyphosis; and Type IV, global kyphosis. According to different sagittal alignments, Type III can further be divided into three subtypes: IIIA, with smooth kyphosis of thoracic and upper lumbar; IIIB, like a clasp knife, with a flat thoracic and lumbar angle; and IIIC, with bigger thoracic kyphosis and lumbar lordosis. The thoracolumbar kyphosis angle (°) of the three subtypes were −23.61 ± 5.37, −25.40 ± 7.71, and −40.01 ± 8.40, respectively. Lumbar lordosis was correlated with thoracic kyphosis (IIIA, r = −0.600, *p* = 0.005; IIIB, r = −0.312, *p* = 0.046; IIIC, r = −0.657, *p* = 0.015), and correlated with sacral slope (IIIA, r = 0.537, *p* = 0.015; IIIB, r = 0.654, *p* = 0.000; IIIC, r = 0.578, *p* = 0.039). All spinopelvic parameters were compared between the Sagittal Balance group and the Sagittal Imbalance group, and only the thoracolumbar kyphosis angle showed statistical difference (t = −2.247, *p* = 0.028). Conclusions: The common characteristics of thoracolumbar junctional kyphosis were found to be a bigger thoracolumbar junctional angle and vertex of kyphosis located in the thoracolumbar junction (T10-L2). Despite TLJK, a change in the thoracic angle was still important to maintain sagittal balance. The thoracolumbar junction plays an important role in sagittal alignment and balance.

## 1. Introduction

The S-shaped curve of the human spine in the sagittal plane plays a crucial role in maintaining balance and stability while minimizing the energy consumption of the lower back muscles [1]. Epidemiological and clinical studies have demonstrated the importance of sagittal balance in preventing spinal dysfunction and evaluating surgical outcomes [2]. The aging spine is characterized by degenerative disc diseases, facet joint arthritis, and the atrophy of extensor muscles, resulting in progressive kyphosis and sagittal disbalance [3]. Takemitsu et al. [4] suggested that “lumbar degenerative kyphosis (LDK) was defined as a kyphosis or a marked loss of lordosis in the lumbar spine, caused by degenerative changes”. LDK is a common disease in middle-aged and elderly women and is related to unhealthy lifestyles, the long-term bending of the waist, and heavy physical labor [5].

LDK is classified into four types of spinal curvatures [4]: Type 1, little lumbar lordosis and marked loss of thoracic kyphosis; Type 2, slight lumbar kyphosis combined with slight lordosis in the thoracic region; Type 3, increased lumbar kyphosis with varying degrees of thoracic lordosis; and Type 4, enlarged thoracic kyphosis that extends downward through the lower lumbar region. Furthermore, Jang et al. [6] divided LDK patients into a thoracic compensatory group and a decompensatory group according to the thoracolumbar junctional angle. In this research, the sagittal thoracic compensated group with a flat or lordosis thoracolumbar junctional angle included a lower lumbar type, a middle lumbar type, and a flat lumbar type, while the sagittal thoracic decompensated group with kyphosis of the thoracolumbar junctional angle was of a global type. This classification could reflect the characteristics of LDK comprehensively and objectively as there is consideration of the relationships between the lumbar and thoracic spine regions.

However, in clinical practice, patients with thoracolumbar junctional kyphosis have attracted more attention, many of whom do not present with LDK. As the transitional area between the lower thoracic spine and the upper lumbar spine, the thoracolumbar junction (TLJ) should play an important role in thoracolumbar/lumbar degenerative kyphosis and sagittal balance. The existing definition and classification of LDK might not represent degenerative sagittal deformity comprehensively. In this research, we aimed to propose a novel classification of thoracolumbar/lumbar degenerative kyphosis and further specify the key roles of the thoracolumbar junction for sagittal alignment and balance.

## 2. Materials and Methods

This study was conducted under the Declaration of Helsinki and was approved by the Ethics Committee of Peking University People’s Hospital (No. 2016PHB186-01). All patients signed written informed consent forms before participating in our study.

Patients with sagittal imbalance due to compression fractures were excluded. Complete radiographic evaluations of the patients with suspected sagittal imbalances were performed using a full-length 36-inch standing lateral radiograph of the entire spine, with the arms held at 60° of forward flexion and the hips and knees fully extended.

**Figure 1:**
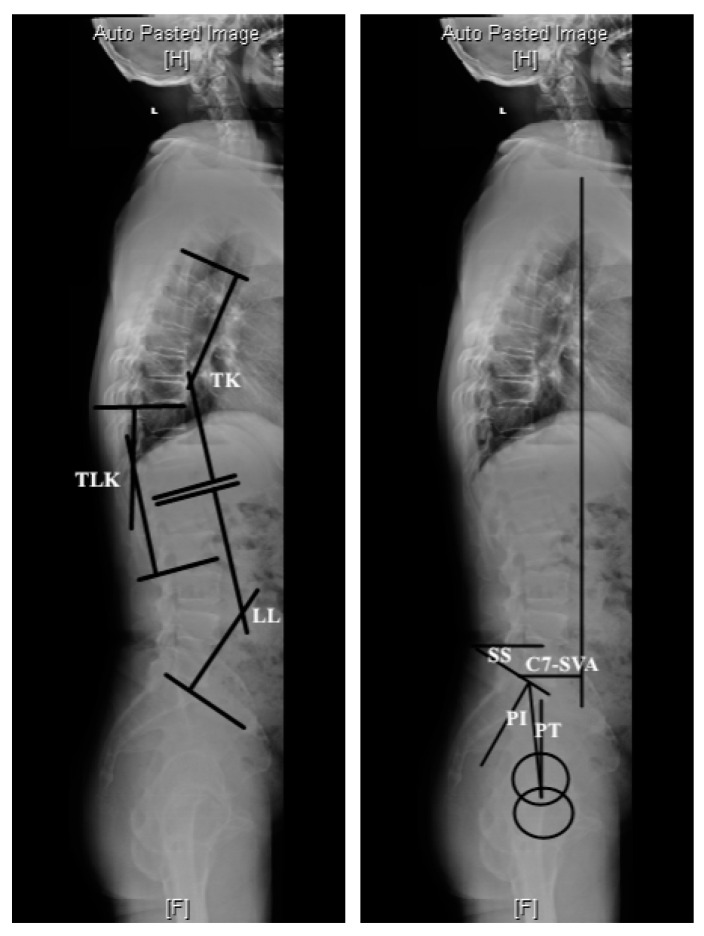
Methods of measurement of sagittal spinopelvic parameters. TLK: thoracolumbar kyphosis; LL: lumbar lordosis; TK: thoracic kyphosis; PI: pelvic incidence; PT: pelvic tilt; SS: sacral slope; C7-SVA: C7-sagittal vertical axis.

As shown in Figure 1, sagittal balance was determined by measuring the sagittal vertical axis (SVA) with a plumb line from the center of the C7 vertebral body to the posterior sacral prominence on the lateral radiograph. A regional sagittal modifier was included to describe each of the three regions of the spine: lumbar lordosis (LL), thoracolumbar kyphosis (TLK), and thoracic kyphosis (TK). The LL was measured from the L1 superior endplate to the S1 superior endplate using the Cobb method. The TLK was measured from the T10 superior endplate to the L2 inferior endplate. The main TK was measured from the T4 superior endplate to the T12 inferior endplate. In terms of TK, TLK, and LL, lordosis was defined as positive and kyphosis as negative.

Pelvic incidence (PI), sacral slope (SS), and pelvic tilt (PT) were measured in each whole spine lateral view. PI was defined as the angle between the line perpendicular to the sacral plate and the line connecting the midpoint of the sacral plate to the bicoxofemoral axis. SS was the angle between the S1 superior endplate and the horizontal line. PT was defined as the angle between the vertical line originating at the center of the bicoxofemoral axis and the line drawn between the same point and the middle of the superior endplate of S1.

Data of LL, TK, and TLK of the three subtypes were compared by independent two-sample *t*-test, and Pearson correlation analyses were used to identify linear relationships between lumbar curve and thoracic curve or sacral angle. All spinopelvic parameters were compared between two groups by independent two-sample *t*-test or Mann–Whitney U-test. Statistical analysis was performed using SPSS 20.0 K (SPSS Inc., Chicago, IL, USA). Statistical significance was defined as *p* < 0.05.

## 3. Results

We enrolled 133 consecutive patients with degenerative spinal kyphosis from September 2016 to March 2019 in our department, and collected data of sagittal parameters. These patients consisted of 43 males and 90 females, averagely aged 67.8 ± 7.63 years old. We summed up the experience of predecessors and added our own insights.

**Figure 2:**
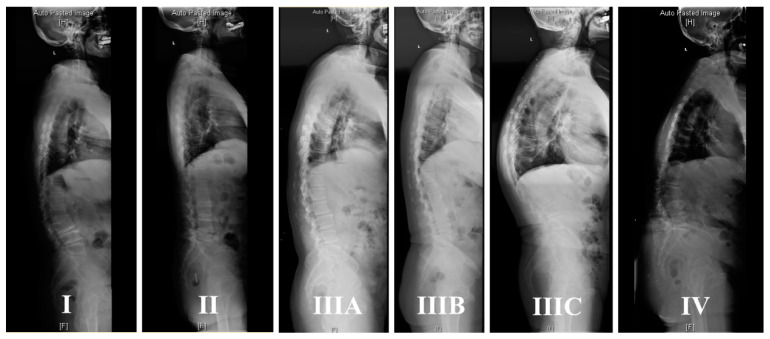
The classification of thoracolumbar/lumbar degenerative kyphosis. Type I, lumbar kyphosis; Type II, degenerative flat back; Type III, thoracolumbar junctional kyphosis (IIIA, IIIB, IIIC); and Type IV, global kyphosis.

Thoracolumbar/lumbar degenerative kyphosis was divided into four types (Figure 2): Type I, lumbar kyphosis, 12 patients (9.02%); Type II, degenerative flat back, 43 patients (32.33%); Type III, thoracolumbar junctional kyphosis, 63 patients (47.37%); and Type IV, global kyphosis, 15 patients (11.28%).

**Figure 3:**
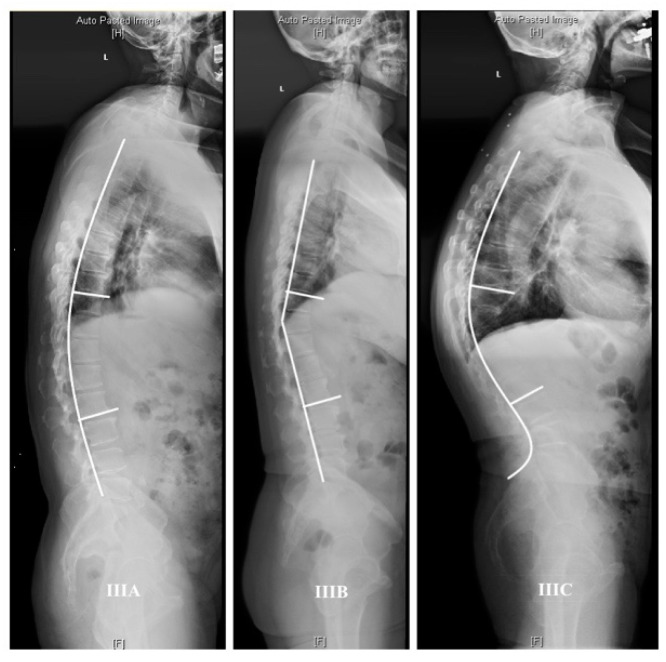
Three subtypes of thoracolumbar junctional kyphosis. IIIA, “(” shape, with smooth kyphosis of thoracic and upper lumbar; IIIB, “<” shape, like a clasp knife, with a flat thoracic and lumbar angle; IIIC, “S” shape, with bigger thoracic kyphosis and lumbar lordosis.

The common characteristics of Type III were found to be a bigger thoracolumbar junctional angle and vertex of kyphosis located in the thoracolumbar junction (T10-L2). According to sagittal alignment, Type III can furtherly be divided into three subtypes (Figure 3): IIIA, 20 patients, “(” shape, with smooth kyphosis of thoracic and upper lumbar; IIIB, 30 patients, “<” shape, like a clasp knife, with a flat thoracic and lumbar angle; and IIIC,13 patients, “S” shape, with bigger thoracic kyphosis and lumbar lordosis.

**Figure 4:**
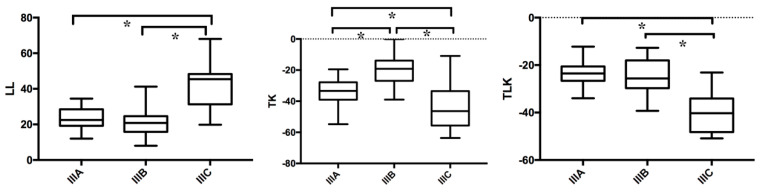
Data of LL, TK, and TLK of three subtypes IIIA, IIIB, and IIIC were compared by independent two-sample *t*-test. * *p* < 0.05. LL: lumbar lordosis; TK: thoracic kyphosis; TLK: thoracolumbar kyphosis.

Data of LL, TK, and TLK of three subtypes were listed and compared by independent two-sample *t*-test. The thoracolumbar kyphosis (TLK) angle (°) of the subtypes IIIA, IIIB, and IIIC were −23.61 ± 5.37, −24.96 ± 6.63, and −40.01 ± 8.40, respectively. Lumbar lordosis (LL) of the subtypes IIIA, IIIB, and IIIC were 23.29 ± 5.90, 20.79 ± 7.55, and 43.18 ± 13.57, respectively. Thoracic kyphosis (TK) of the subtypes IIIA, IIIB, and IIIC were −34.18 ± 8.31, −20.50 ± 10.03, and −43.92 ± 14.80, respectively. As shown in Figure 4, significant differences of LL were observed in IIIA versus IIIC and IIIB versus IIIC; significant differences of TK were observed among three subtypes; significant differences of TLK were observed in IIIA versus IIIC and IIIB versus IIIC.

**Figure 5:**
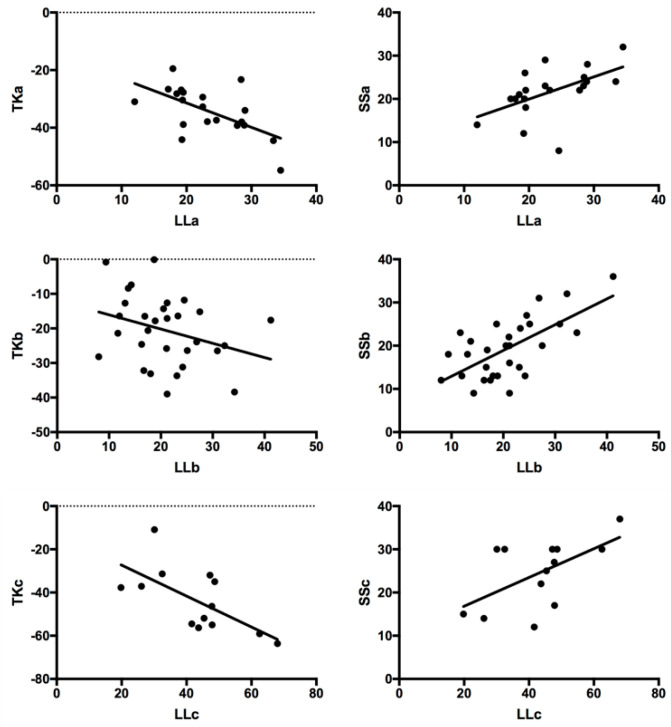
Correlation analysis between spinopelvic parameters in three subtypes. LL was all correlated with TK (IIIA, r = −0.600, *p* = 0.005; IIIB, r = −0.312, *p* = 0.046; IIIC, r = −0.657, *p* = 0.015), and all correlated with SS (IIIA, r = 0.537, *p* = 0.015; IIIB, r = 0.654, *p* = 0.000; IIIC, r = 0.578, *p* = 0.039).

Figure 5 displays the correlation coefficients between parameters in three subtypes. For each subtype, LL was all correlated with TK (IIIA, r = −0.600, *p* = 0.005; IIIB, r = −0.312, *p* = 0.046; IIIC, r = −0.657, *p* = 0.015), and all correlated with SS (IIIA, r = 0.537, *p* = 0.015; IIIB, r = 0.654, *p* = 0.000; IIIC, r = 0.578, *p* = 0.039).

Then, 63 patients of Type III were divided into two groups according to C7-SVA: the Sagittal Balance group (C7-SVA < 50 mm, 38 patients) and the Sagittal Imbalance group (C7-SVA ≥ 50 mm, 25 patients). All spinopelvic parameters were compared between two groups, and we found that only the TLK showed statistical differences (t = −2.247, *p* = 0.028) (Table 1). We speculated that thoracolumbar kyphosis angle played an important role for sagittal alignment and balance.

## 4. Discussion

In the process of an aging society, prevalence of thoracolumbar/lumbar degenerative kyphosis gradually increases. With osteoporosis, intervertebral disc degeneration, and muscle dysfunction [7], spinal degenerations and deformities continue to progress, significantly affecting the quality of life in old patients. In our study, we not only deepened our understanding and recognition of previous classifications of lumbar degenerative kyphosis through the review of relevant cases, but, more importantly, we proposed the characteristics of thoracolumbar junctional kyphosis and preliminarily explored and confirmed the roles of thoracolumbar junction in the occurrence and development of spinal degenerative kyphosis. In addition, through the study of compensatory mechanisms of sagittal balance, it is proposed that changes in thoracic curve play an important role in maintaining sagittal balance for patients with thoracolumbar junctional kyphosis. 

### 4.1. Normal Sagittal Alignment

Sagittal balance is an important concept for understanding and treating spinal diseases [8]. It originated from the pelvic index proposed by Duval-Beaupère et al., which included pelvic incidence (PI), pelvic tilt (PT), sacral slope (SS), and PI = PT + SS. PI increases with age and eventually becomes a constant anatomical parameter in adulthood. More importantly, lumbar lordosis (LL) and thoracic kyphosis (TK) extremely depend on PI. The PI value has certain clinical significance: patients with larger PI also have larger lumbar lordosis and higher shear stress; On the contrary, patients with smaller PI have relatively lower shear stress. Therefore, patients with larger PI are prone to have lumbar spondylolysis. Sagittal compensatory mechanisms largely depend on PI: the compensatory capacity of low PI is small, while that of high PI is large.

In 2005, Pierre Roussouly [9] showed the classification of the normal variation in the sagittal alignment of the human lumbar spine and pelvis in the standing position, including four types: Type 1, low SS, long thoracolumbar kyphosis and short lumbar lordosis curve; Type 2, low SS, flat back; Type 3, SS is relatively high, thoracic kyphosis and lumbar lordosis are almost equal; Type 4, SS is very high, presenting a larger lumbar lordosis and a shorter thoracic kyphosis. This classification helps to determine the high stress areas of the spine: a greater curvature of the lumbar leads to a greater stress on the posterior structures (like facet joints); a lower of the position of the lumbar curvature or the flat back leads to a greater stress on the intervertebral disc. 

Although in our research, sagittal morphology was similar between subtype IIIA and Roussouly type 1, or between subtype IIIC and Roussouly type 4, the common characteristics of our patients were found to have a bigger thoracolumbar junctional angle and vertex of kyphosis located in the thoracolumbar junction (TLJ, T10-L2), based on the fact that the normal apex of thoracic kyphosis in healthy adults is located at T5–T7 [10].

### 4.2. Thoracolumbar Junctional Kyphosis

Previous studies about thoracolumbar kyphosis have mainly focused on ankylosing spondylitis, Scheuermann’s disease, and thoracolumbar fractures [11,12,13], as well as risk factors for proximal junctional kyphosis after long segment fixation surgery [14]. Gao et al. [15] believed that the preoperative application of digital intelligent software Surgimap Spine contributed to the design and treatment of thoracolumbar kyphosis in ankylosing spondylitis patients, and the clinical efficacy was satisfactory. Anterior combined posterior surgery was once considered as the gold standard for surgical treatment of Scheuermann’s disease. In recent years, with the application of posterior osteotomy and pedicle screws, posterior surgery has gradually become the preferred surgical method due to its advantages of shorter surgical time, less blood loss, fewer complications, and more satisfactory therapeutic effects. However, the complications of posterior surgery, such as proximal junctional kyphosis (PJK) and distal junctional kyphosis (DJK), have gradually attracted attention [12]. Thoracolumbar osteoporotic vertebral compression fractures with kyphosis deformity are often seen in elderly patients with multiple preoperative comorbidities and more postoperative complications, including displacement and loosening of bone cement and internal fixation. Reasonable treatments should be adopted according to the patient’s specific situation, such as percutaneous vertebral plasty (PVP), percutaneous kyphoplasty (PKP), or various osteotomy and corrective surgeries. Currently, there is great controversy over surgical indications and methods. The presence of nerve compression, the shape and degree of kyphosis, the degree of anterior and middle column collapse of the injured vertebra, and loading capacity are key factors affecting the selection of surgical procedures. For patients with relatively stable arc-shaped kyphosis and without nerve compression, PKP could be used to correct kyphosis [16]. However, for patients suffering from severe kyphosis and instability, corrective surgery should be considered to improve severe back pain [17]. When formulating diagnosis and treatment for patients with degenerative spinal diseases, comprehensive consideration should be given. Patients with large or small preoperative TK should be treated carefully and comprehensive analysis of parameters such as SVA, PI, and LL should be included [13]. When determining the degree of sagittal correction in patients, age-related soft tissue and spinal degeneration could not be ignored, and it is necessary to make surgical plans based on age. When determining the position of upper instrumented vertebra (UIV) or lower instrumented vertebra (LIV), the appropriate fusion vertebra should be selected based on the different degrees of sagittal imbalance and the overall biomechanical effects of the spine. However, there is still a lack of clear evidence to prove the positive effects of preventive bone cement reinforcement on adjacent vertebra [18].

In our previous clinical research [19], we found that thoracolumbar junctional kyphosis often accompanied lumbar degenerative kyphosis. However, former classifications might be not comprehensive enough, given the truth that some patients with thoracolumbar kyphosis are not combined with marked loss of lumbar lordosis. As we know, the thoracolumbar junction, where increasing torsional stiffness and specifically directed shear loads of the spine have been observed, is the transitional area of biomechanics and morphology [20]. Vertebral compression fractures and proximal junctional kyphosis (PJK) following spine surgery often occur in this area [21]. Therefore, we have preliminarily demonstrated that thoracolumbar junctional kyphosis might be generated by special characteristics of morphology and biomechanics of the TLJ, not just following the primary degenerations of lumbar spine. In view of the above, both surgical strategy for patients with thoracolumbar kyphosis and regulating mechanisms of sagittal balance needed more thorough discussions.

### 4.3. Surgical Strategy

The surgical indications for thoracolumbar/lumbar degenerative kyphosis are as follows [22]: (1) conservative treatment failure and severe lower back pain; (2) progressive worsening of neurogenic symptoms, or severe deformities that affect quality of life; (3) severe sagittal imbalance; (4) rapid progression of spinal deformities. The formulation of surgical strategies mainly involves appropriate osteotomy methods, selection of UIV (upper instrumented vertebra) and DIV (distal instrumented vertebra), and so on [23].

**Figure 6:**
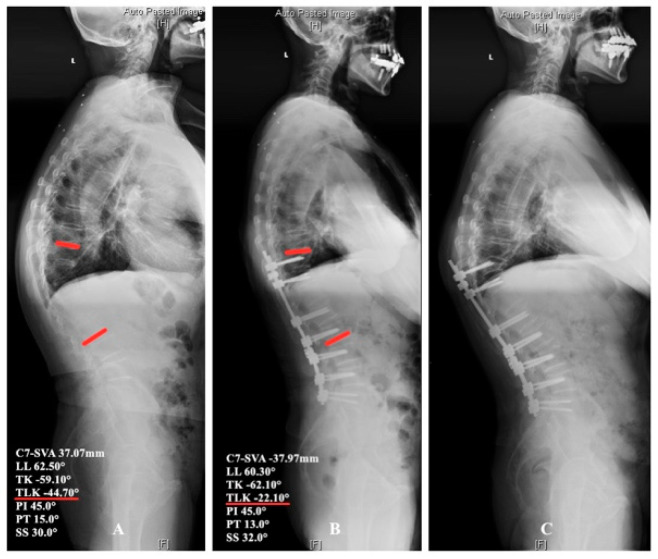
Proximal junctional failure for a patient of subtype IIIC. (**A**) Male, 72 years old, a typical patient of subtype IIIC. (**B**) This patient underwent T10-L5 fusion and L3/4 partial osteotomy, without osteotomy at the apex of kyphosis T11. After the surgery, T9 became the new apex, TLK became −22.10° from −44.70°, and C7-SVA became −37.97 mm from 37.07 mm, without significant change of LL, TK, and SS. (**C**) This patient received revision surgery because of fracture and instrumentation failure at upper instrumented vertebrae 5 months after surgery. For this patient, appropriate osteotomy around apex of kyphosis should be finished, UIV should exceed thoracolumbar junction, and latrogenic apex of kyphosis should be avoided.

Correct selection of fusion segments is the key factor for perfect surgical outcomes. Inappropriate upper instrumented vertebra (UIV) might result in unsatisfactory surgical correction, worse deformity, or loss of correction, and even revision surgery [24]. UIV should be stable, neural, and horizontal vertebra in the coronal and sagittal plane, without obvious degeneration and kyphosis at adjacent segments [25]. Silva and Lenke [26] recommended the Lenke–Silva graded treatment system based on lumbar lordosis and global balance for degenerative scoliosis patients. Kim et al. [27] advised that for patients with thoracolumbar/lumbar kyphosis, we should select UIV and osteotomy location according to Takemitsu’s classification. Our classification of the sagittal alignment of thoracolumbar/lumbar degenerative kyphosis was beneficial for surgical strategy. Firstly, patients with sagittal imbalance often have obvious symptoms of severe lumbar stenosis, and the aim of surgery was decompression of spinal cord, neurolysis of compressed nerve root, termination of deformity development, and rebuilding spinal balance. Therefore, for patients with obvious symptoms of lumbar stenosis and acceptable symptoms related with local kyphosis, limited fusion was the reasonable choice. Secondly, it is essential for patients with severe thoracolumbar kyphosis, especially subtypes IIIB and IIIC, to undergo appropriate osteotomy around apex of kyphosis. Thirdly, UIV should exceed thoracolumbar junction, and latrogenic apex of kyphosis should be avoided. It is important for spinal surgeons to prevent proximal junctional kyphosis (PJK) or proximal junctional failure (PJF) and instrumentation-related complications (Figure 6). The prevalence of PJK was 8.1–46%, and the main risk factors included excessive correction of sagittal imbalance, proximal junctional fracture, geriatric, high body mass index, and lower fixation to sacrum [28]. It is worth mentioning that age-adjusted alignment goals are necessary because older patients are inherently more kyphotic and do not require so much correction [29]. Lafage et al. [30] showed that PJK might be a component of the compensatory mechanisms for sagittal overcorrection and patients with PJK often have smaller postoperative PI-LL mismatches indicate overcorrection during surgery as the main culprit. Mauro et al. [31] investigated spinopelvic parameters and reported that PJK increased in patients who had higher preoperative thoracic kyphosis, proximal junction angulation, pelvic incidence (PI), and LL. Thoracic hyperkyphosis is also regarded as a risk factor of PJK and PJF. 

It should be emphasized that for the treatment of spinal deformities, the surgical goal is to alleviate the patient’s clinical symptoms, rather than correcting imaging parameters. When formulating the surgical strategies, these principles should be followed: (1) For the treatment of degenerative diseases, the fixed segments should be shorter rather than longer; (2) For mild deformities, neural decompression should be the main treatment, and preventive surgery for correction is not recommended; (3) For patients with high surgical risks, such as multiple preoperative comorbidities, severe osteoporosis, sarcopenia, and neuromuscular diseases, long segment fixation surgery should be avoided as much as possible; (4) Long segment correction surgery is only considered when it is clear that the patient’s severe clinical symptoms are related to the deformity, and after sufficient communication with the patient for the surgical risks.

### 4.4. Compensatory Mechanisms for Sagittal Balance

For the maintenance of sagittal balance, reduction of thoracic kyphosis, intervertebral hyperextension, retrolisthesis, pelvis back tilt, knee flessum, and ankle extension were the main mechanisms described in the literature [32]. The basic concept of these compensatory mechanisms was to extend adjacent segments of the kyphotic spine, allowing for compensation of anterior translation of the axis of gravity. The sagittal balance and imbalance are dynamic processes [33]. As the deformity progresses, the human body tends to activate more compensatory mechanisms to maintain the sagittal balance. When all mechanisms reach their maximum and the sagittal balance cannot be maintained, the body enters a decompensated state: the overall center of gravity shifts forward and the patient needs to maintain an upright position with the help of external objects (such as crutches). At this time, the cervical spine is mostly in a forward convex to maintain a horizontal line of sight [34]. At present, it is believed that compensatory mechanisms for sagittal balance are chainlike. The abnormal initiating factors of sagittal plane sequences are loss of lumbar lordosis (LL) and mismatch between pelvic incidence (PI) and LL: (1) To compensate for the loss of LL, the posterior rotation of the pelvis is initiated first, manifested as an increase in pelvic tilt (PT) and a decrease in sacral slope (SS) [35]. (2) If the pelvic posterior rotation is not sufficient to compensate, thoracic compensation will be initiated: the reduction of thoracic kyphosis or even thoracic lordosis. (3) If the above compensations still cannot meet the requirements, lower limb compensation should be initiated: knee flexion, which may not always be compensated, and some patients may exhibit sagittal decompensation. The above compensatory order is based on a cross-sectional study with a large sample size and is not a conclusion of longitudinal follow-up, so it is only applicable to general situations. The order of compensation may vary for each patient due to differences in the location of the deformity (such as the posterior convexity vertex). 

As for compensatory mechanisms for sagittal balance, our research investigated some findings different from previous studies. In our opinion, change of thoracic angle and pelvis retroversion were both important for sagittal balance in patients with thoracolumbar kyphosis. Some experts [36] proposed that when the spine is rigid (aged kyphotic and ankylosis), there is no possibility for the patient to reduce the magnitude of the thoracic curve, and the only compensatory mechanism in the pelvis area is pelvis back tilt (also called pelvis retroversion). For patients with thoracolumbar kyphosis, decreasing sacral slope showed that pelvis retroversion was anticipated in compensatory mechanisms of sagittal balance; but relatively small pelvis incidence indicated limited capacity of pelvis back tilt, so thoracic compensation seemed to be more important. 

There were several potential limitations in this study. Firstly, the number of patients was relatively small. Larger sample size would improve study robustness. Secondly, future guides for surgical therapy should be based on biomechanical studies, in particular, of older patients with sagittal disbalance. 

In conclusion, thoracolumbar junction is important for sagittal alignment and balance. The common characteristics of thoracolumbar junctional kyphosis were found to be a bigger thoracolumbar junctional angle and vertex of kyphosis located in thoracolumbar junction (T10-L2). Despite thoracolumbar junctional kyphosis, change of thoracic angle was still important to maintain sagittal balance. Fully understanding the thoracolumbar junction’s effects on sagittal alignment and balance will contribute to make reasonable surgical strategies for degenerative spinal kyphosis.

## Figures and Tables

**Table 1:** Comparison of spinopelvic parameters between the Sagittal Balance group and the Sagittal Imbalance group.

Spinopelvic Parameters	Sagittal Balance Group	Sagittal Imbalance Group	Statistical Values	*p*
TLK	−29.674 ± 10.050	−24.540 ± 6.660	t = −2.247 *	0.028
LL	24.550 (18.850, 35.950)	21.100 (17.400, 28.150)	Z = −1.658 ^†^	0.097
TK	−29.474 ± 15.740	−29.984 ± 11.648	t = 0.139 *	0.890
PI	38.553 ± 9.217	42.360 ± 9.380	t = −1.593 *	0.116
PT	16.500 (13.000, 25.250)	20.000 (13.000, 25.000)	Z = −0.493 ^†^	0.622
SS	20.053 ± 7.819	22.840 ± 4.947	t = −1.733 *	0.088

The data are shown as mean ± SD or median (Q1, Q3). * Independent two-sample *t*-test; ^†^ Mann–Whitney U-test. TLK: thoracolumbar kyphosis; LL: lumbar lordosis; TK: thoracic kyphosis; PI: pelvic incidence; PT: pelvic tilt; SS: sacral slope; SD: standard deviation.

## Data Availability

The data that support the findings of this study are available from our hospital but restrictions apply to the availability of these data, which were used under license for the current study, and so are not publicly available. The data are, however, available from the authors upon reasonable request and with permission of our hospital. Interested parties may contact the first author for further information.
